# Dietary total antioxidant capacity (TAC) among candidates for coronary artery bypass grafting (CABG) surgery: Emphasis to possible beneficial role of TAC on serum vitamin D

**DOI:** 10.1371/journal.pone.0208806

**Published:** 2018-12-12

**Authors:** Mahdieh Abbasalizad Farhangi, Mahdi Najafi

**Affiliations:** 1 Nutrition Research Center, Tabriz University of Medical Sciences, Tabriz, Iran; 2 Drug Applied Research Center, Tabriz University of Medical Sciences, Tabriz, Iran; 3 Department of Research, Tehran Heart Center, Tehran University of Medical Sciences, Tehran, Iran; 4 Cardiac Outcome Research and Education (CORE), Universal Scientific Education and Research Network (USERN), Tehran, Iran; University of Bologna, ITALY

## Abstract

**Aims:**

Recently, the clinical importance of total antioxidant capacity (TAC) and its protective role against several chronic diseases like cardiovascular disease, osteoporosis and several types of cancers has been reported. However, its association with cardio-metabolic risk factors among patients candidate for coronary artery bypass graft surgery (CABG) has not been evaluated yet. CABG is associated with increased oxidative stress and free radicals; so, the current study was aimed to evaluate the potential association of TAC with cardiovascular risk factors among patients candidate for CABG.

**Methods and materials:**

In the current cross-sectional study, 454 patients aged 35–80 years as candidates of CABG and hospitalized in Tehran Heart Center were enrolled. Anthropometric and demographic characteristics were obtained from all participants. Total dietary antioxidant capacity (TAC) was calculated according to the findings of semi-quantitative food-frequency questionnaire (FFQ). Biochemical parameters including serum lipids, albumin, creatinine, HbA1C, C-reactive protein (CRP), lipoprotein (a), creatinine, blood urea nitrogen (BUN) and serum vitamin D concentrations were also assessed by commercial laboratory methods.

**Results:**

Male patients in the top quintiles of TAC had significantly lower prevalence of hypertension (35.1% vs 45.9%). Moreover, male patients at fifth quintile of TAC were 2% more serum vitamin D concentrations, 3% lower serum cholesterol concentrations compared with lowest quintile. Top quintiles of TAC make patients more likely to have low hematocrit and high serum albumin concentrations compared with lowest quintiles (P < 0.05). However, in female participants, only serum creatinine concentration was in negative association with TAC. In comparison of clinical parameters, females in the fifth quintile of TAC had 9% higher EF compared with patients in the first quintile (P = 0.021).

**Conclusion:**

To our findings, higher dietary antioxidant capacity was associated with lower prevalence of hypertension, lower hematocrit, total cholesterol and higher albumin and vitamin D concentrations. Therefore, high dietary TAC could be considered as a potent protective tool against cardio-metabolic risk factors in patients CABG candidate for especially in male patients.

## Introduction

Coronary artery bypass grafting surgery (CABG), as a category of coronary revascularization, is one of the most frequent procedures performed and annually about 50000 open-heart surgeries are performed in Iran, 50–60% of which is allocated to CABG [1]. The CABG was first introduced in the 1960s with the aim of offering symptomatic relief, improved quality of life, and increased life expectancy in patients with coronary artery disease (CAD) [2–3]. The surgery major indicators are over 50% left main coronary artery stenosis or over 70% stenosis of the proximal left anterior descending (LAD) and proximal circumflex arteries [4]. According to the world health organization report, cardiovascular diseases (CVDs) are the number one cause of death globally: more people die annually from CVDs than from any other cause. An estimated 17.7 million people died from CVDs in 2015, representing 31% of all global deaths. Of these deaths, an estimated 7.4 million were due to coronary heart disease and 6.7 million were due to stroke [5]. In Iran, also CAD is a major cause of mortality and morbidity and accounts for nearly 50 percent of all deaths per year [6].

It is well known that oxidative stress is involved in the pathogenesis and development of coronary artery disease and atherosclerosis; increased serum free radicals include reactive intermediates of oxygen—reactive oxygen species metabolism (ROMs), such as superoxide radicals (O2-·) hydroperoxyl (HO2·) and hydroxyl (OH·) and hydrogen peroxide (H_2_O_2_) are associated with the extent and severity of CAD and the occurrence of different atherogenic risk factors [7–9]. Most importantly, patients candidate for CABG have increased markers of oxidative stress [10] and the CABG process by itself, triggers the oxidative stress status even during and after surgery [11]; in the study by Cavalca et al increased oxidative stress regarding an impairment in the arginine/nitric oxide pathway and reduced antioxidant level has been reported in candidates of CABG [12–13].

The role of nutritional behaviors and dietary intakes in prevention of CVD or its progression has been studied before; numerous studies suggested the role of healthy dietary choices and improved life style with higher physical activity level [14] and higher intakes of healthy food choices including fruits and vegetables and dietary antioxidants in prevention and treatment of cardiovascular events [15]; moreover, there are multiple studies have investigated the role of single anti-oxidant nutrients as a supplement [16, 17] in improving the cardiovascular disease severity and attenuating the CABG consequences.

Dietary total antioxidant capacity (TAC) reflects all of the antioxidant compounds present in food and the interactions between those compounds [18]. The total antioxidant capacity of diet is inversely associated with cardiovascular diseases [19], heart failure [20] myocardial infarction [21] stroke [22]. However, as mentioned above, studying the potential antioxidant capacity of diet in the pre-CABG patients could be considered as a useful preventive tool to assess the possible need to antioxidants. Moreover, while vitamin D is classically well-known for its classic role in bone homeostasis, recent studies have identified new roles for it. The antioxidant activity of vitamin D has been proved by Wiseman in 1993 while indicating its role in prevention of lipid peroxidation in the cell membrane [23]. Moreover, it has been identified that vitamin D deficinecy is a powerful contributor of cardiovascular deaths; In a cohort study of 3258 participants, death from cardiovascular disease in patients with severe and moderate vitamin D deficiency was 1.8 to 2.5 times more prevalent compared with patients with mild or no vitamin D deficiency [24].

Therefore in the current study we aimed to evaluate the total dietary anti-oxidant capacity and its association with metabolic risk factors of CVD and serum vitamin D concentrations in patients candidate of CABG.

## Materials and methods

### Subjects

The study protocol has been reported elsewhere [25]; participants in the current cross-sectional study were recruited for Tehran Heart Center-Coronary Outcome Measurement (THC-COM) study and were candidates for isolated CABG with cardiopulmonary bypass. Participants in this study were patients admitted to the cardiothoracic ward for CABG surgery at a large heart center in this time period. The sample size calculation has been explained before [26]; briefly, the sample size was calculated using the formula for comparing two proportions: n = [(Zα/2 + Zβ)2 × {(p_1_ (1-p_1_) + (p2 (1-p_2_))}]/ (p_1_—p_2_)^2^ where p_1_ is the proportion of the women with low quality Mediterranean regimen (0.3), p_2_ is the proportion of the men with low quality Mediterranean regimen (0.25), α error = 0.05, and power = 80% (1-β). Accordingly, a 125-subject sample size was determined for the study (125 in each group). We also assumed 20% loss (125 + 25) and as men with CAD are twice as women (150 + 300), the final sample size of 450 was considered for the study [26–28]. Reasons for drop-out or exclusion were incomplete dietary questionnaires (n = 1), and incomplete demographic questionnaires (n = 5). Finally, 454 patients aged 35–80 years completed the study. The details of study procedure and biochemical assays have been provided elsewhere [26]. The study was approved by the ethics committee of Tehran Heart Center, Tehran University of Medical Sciences, Research Undersecretary of Tabriz University of Medical Sciences, and written informed consent was obtained from all of the participants.

### Clinical assessment of patients

Clinical assessment and pre-operative cardiac status was also measured by several variables including: left ventricular ejection- fraction, number of diseased vessels, New York Heart Association (NYHA) functional class and the European system for cardiac operative risk evaluation (EuroSCORE) [29]. EuroSCORE is a simple, additive risk model of perioperative mortality and as a useful predictor of the long term hazard of cardiovascular events leading to death or morbidity after cardiac surgery [30]. It is calculated according to the standard additive methods and was assessed as a continuous variable [31]. NYHA functional classification provides a simple way of classifying the extent of heart failure. It places patients in one of four categories based on how much they are limited during physical activity; Class I denotes normal activity and class IV indicates there are problems such as dyspnea even at rest [32].

### Anthropometric assessments

Anthropometric variables including weight and height were measured and body mass index (BMI) was calculated. Weight was measured while subjects wearing light clothes [33].

### Dietary assessments and calculation of total antioxidant capacity

Total antioxidant capacity of diet (TAC) was calculated based on a 138-item semi-quantitative food frequency questionnaire (FFQ) consisting of a list of foods with standard serving sizes commonly consumed by Iranians. Participants were asked to report how often they consumed each of the food items listed as the number of times per day, per week, per month or per year during the previous year. The reported frequency for each food item was then converted to a daily intake. Portion sizes of consumed foods were converted to grams by using household measures [34]. The questionnaire was previously validated for healthy Iranian population [35]. TAC was calculated using Trolox equivalent according to previous database of the common foods antioxidant capacity estimated by Oxygen Radical Absorbance Capacity (ORAC) assay [36, 37]. The ORAC assay measures the inhibition of peroxyl radical–induced oxidation by the test sample and is expressed as μmol-Trolox equivalent (TE)/100g; Trolox is a potent antioxidant derived from vitamin E [37]. The total antioxidant capacity of diet was calculated by multiplying the average frequency of consumption of each food by ORAC content (μmol TE/100 g) of age-specific portion sizes. Overall, in the 138-item food-frequency questionnaire there were 100 items with ORAC values. Total antioxidant capacity of diet was adjusted for total energy intake with the residual method [38].

### Statistics

Analysis of data was performed by SPSS software (statistical package for social analysis, version 18, SPSS Inc., Chicago, IL, USA). The normality of data was tested by Kolmogorov-Smirnov test. The comparison of discrete and continuous variables between different quartiles of TAC score was performed by Chi- square test and analysis of variance (ANOVA) respectively. Odds ratios and 95% confidence intervals for the association between different TAC quintiles and clinical parameters were estimated using multivariate multinomial logistic regression models adjusting for confounders including age, BMI, educational attainment and presence of diabetes and myocardial infarction. All data are expressed as means ± SD or number and percent. A two-sided *P* value less than 0.05 was considered significant.

## Results

**Fig 1:**
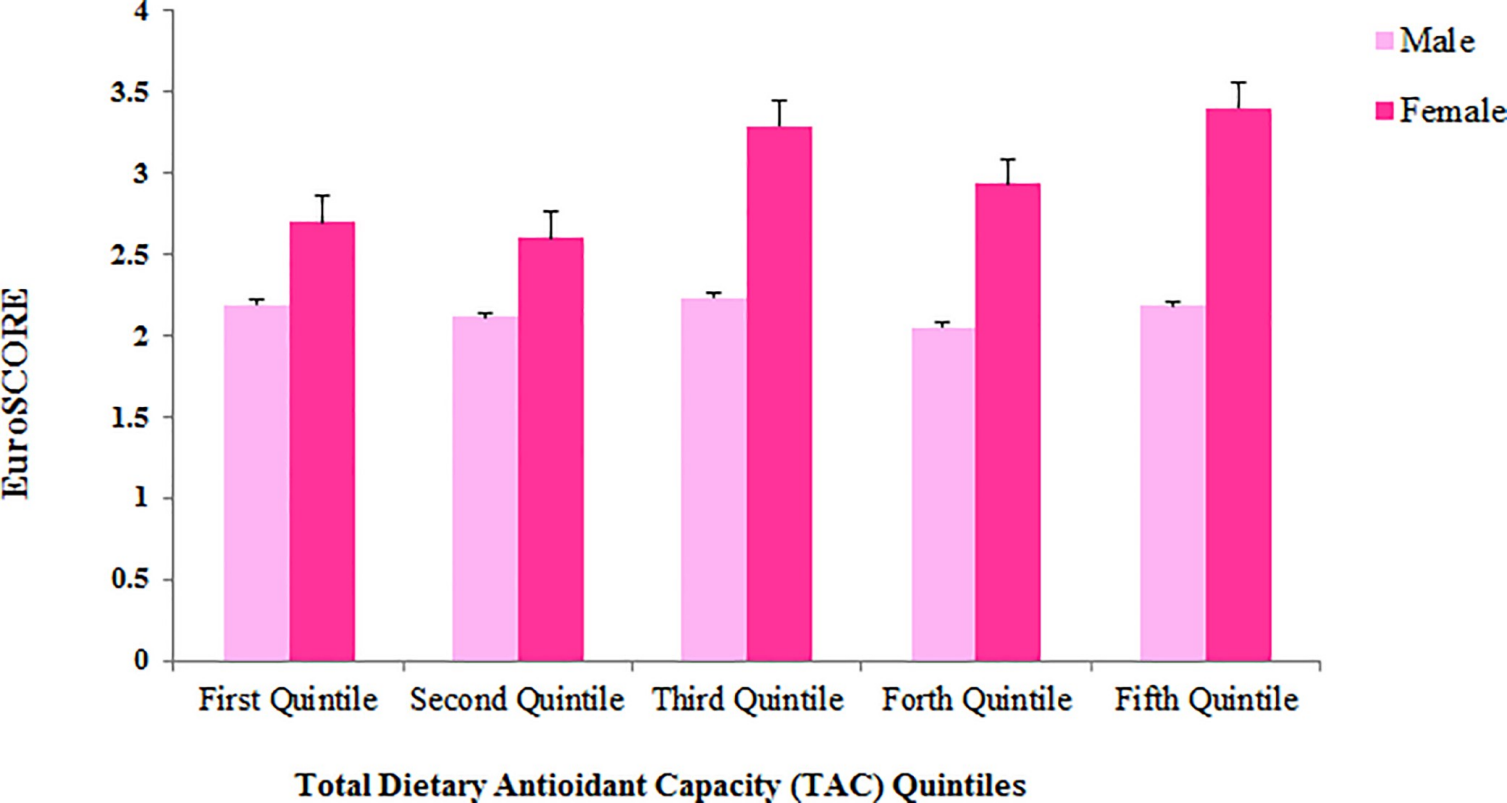
European system for cardiac operative risk evaluation (EuroSCORE) in patients according to TAC quintiles; no significant difference was observed.

**Fig 2:**
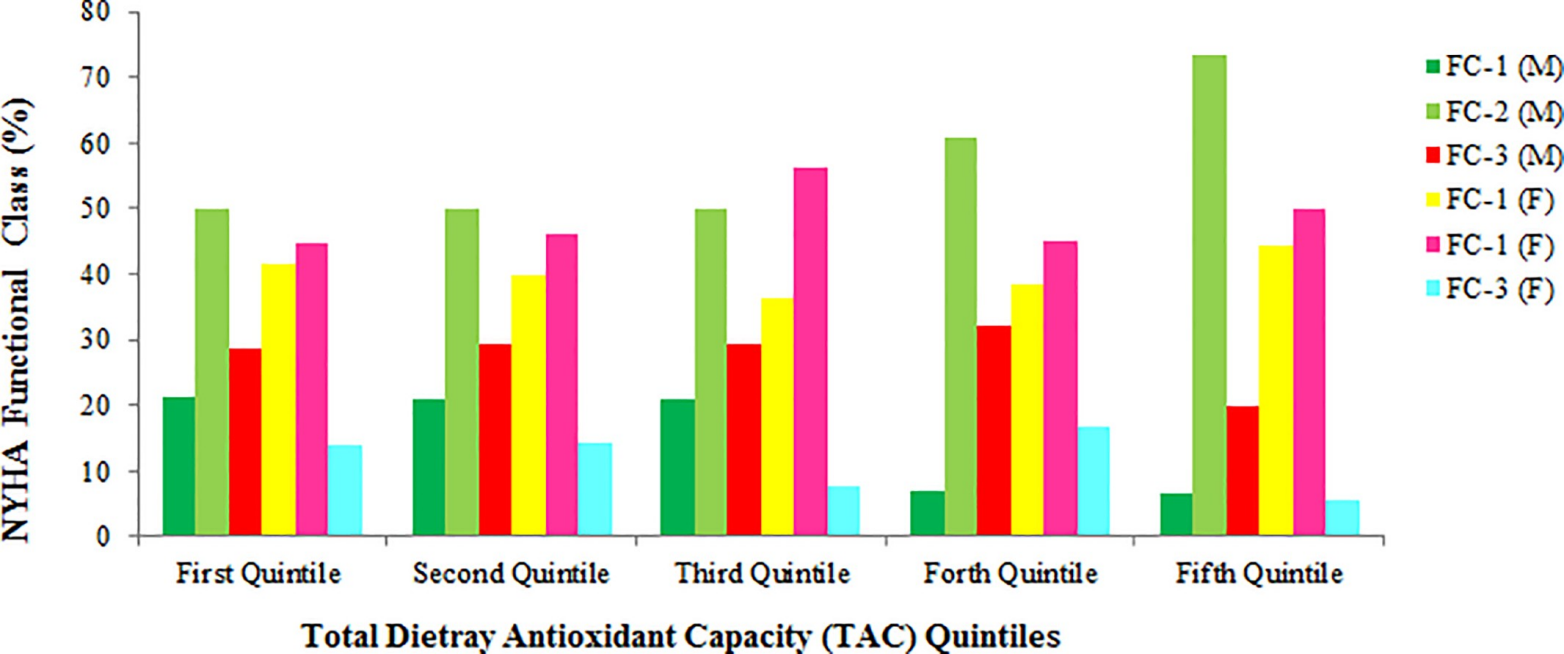
New York Heart Association (NYHA) functional class in patients according to TAC quartiles; no significant difference between different quintiles of TAC has been observed.

**Fig 3:**
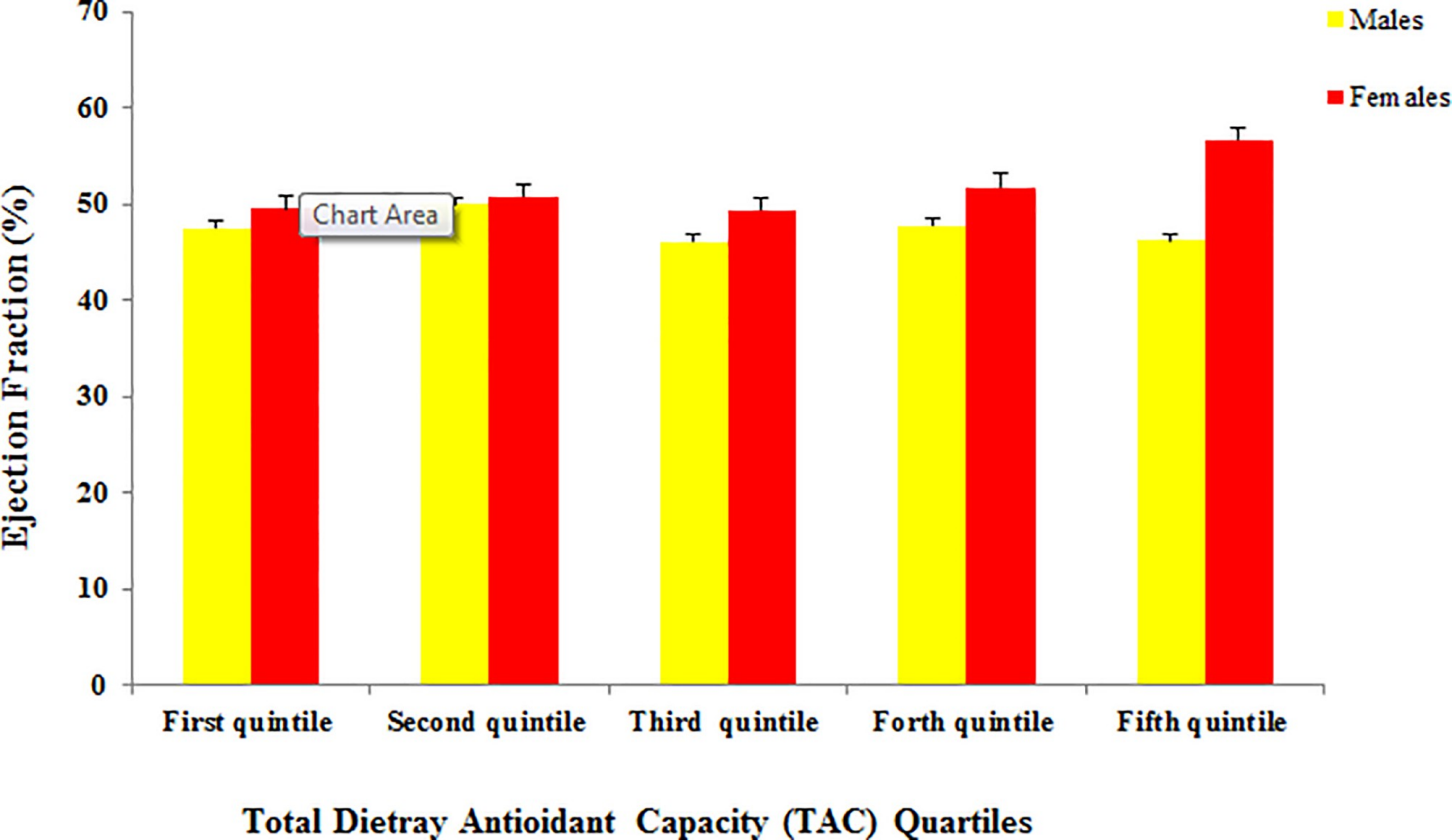
Left ventricular ejection-fraction in patients according to TAC quartiles; female in the fifth quintile of TAC had 9% higher EF compared with patients in the first quintile (P = 0.021).

**Fig 4:**
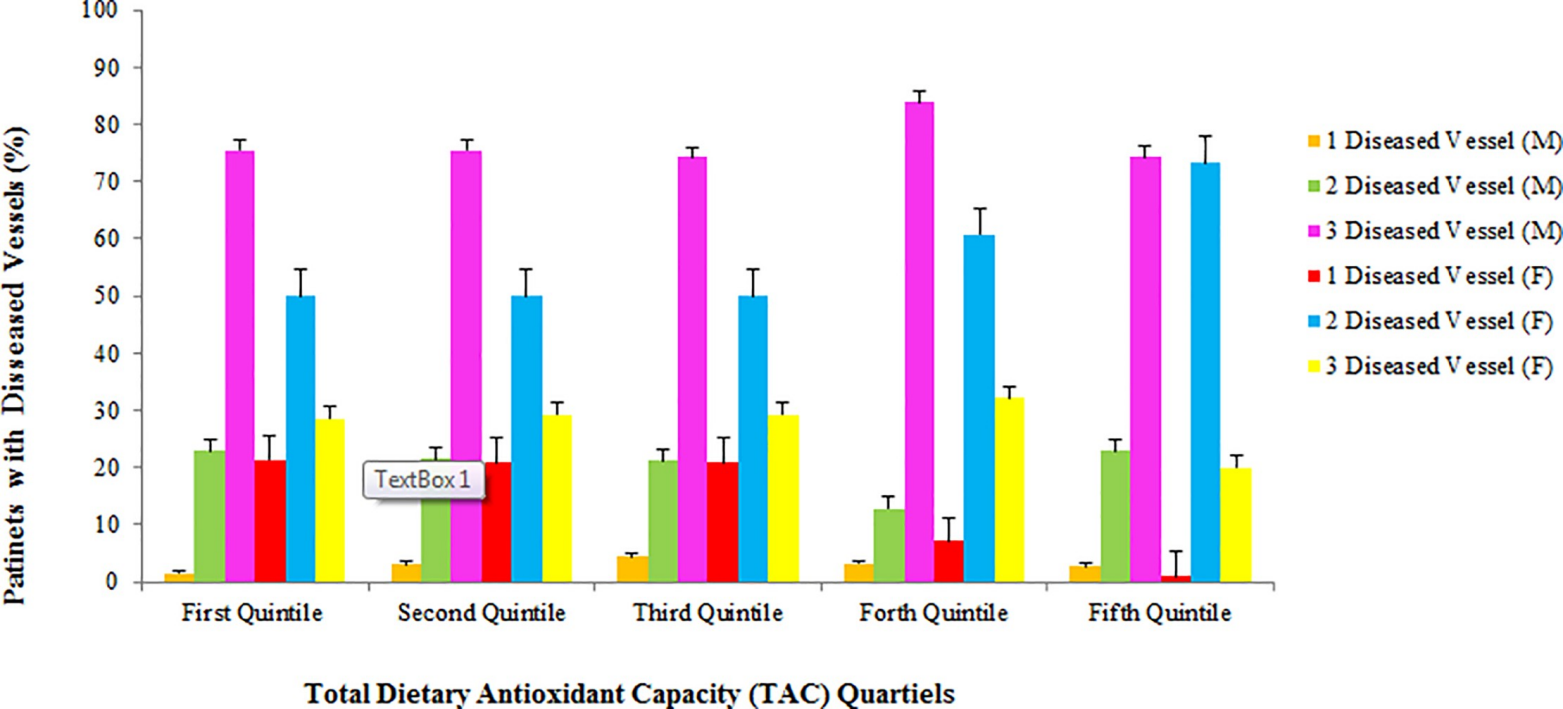
Number of diseased vessels in patients according to TAC quintiles; no significant difference between different quintiles of TAC has been observed.

Table 1 presents the baseline characteristics of male participants in different TAC quintiles. Male patients in the first quintile of TAC had significantly higher prevalence of hypertension compared with other quintiles. Other parameters including age, BMI, presence of MI, diabetes, smoking and hyperlipidemia were not significantly different between patients’ in different TAC quintiles. Moreover, in females (Table 2), none of these parameters were different (P > 0.05). As shown in Table 3, male patients in second and fifth quintile of TAC had 2–3% higher amounts of serum vitamin D compared with first quintile (P <0.05). Moreover, male patients in the second, third, fourth and fifth quintiles, had approximately 10–17% lower hematocrit concentrations compared with first quintile and being in top quintiles of TAC was associated with higher serum albumin concentrations. In female participants (Table 4), however, only serum creatinine concentration was in negative association with TAC. In comparison of clinical parameters in study participants in a gender separated analysis (Figs 1–4), female in the fifth quintile of TAC had 9% higher left ventricular ejection-fraction compared with patients in the first quintile (P = 0.021).

**Table 1:** Baseline characteristics of male patients candidate for CABG according to TAC quintiles.

Quintiles of TAC score						
Variable	1^st^ quintiles	2^nd^ quintiles	3^rd^ quintiles	4^th^ quintiles	5^th^ quintiles	P value
	N = 61	N = 66	N = 66	N = 62	N = 74	
**Age (y)**	60.02±10.51	59.88 ±10.07	57.68±8.61	57.42±9.37	58.50±8.56	0.38
**BMI (kg/m**^**2**^**)**	25.84±3.52	27.17±2.96	26.89±3.69	26.36±3.52	26.86±3.75	0.22
**Diabetic [n (%)]**	20 (32.8)	19 (28.8)	25 (37.9)	23 (37.1)	22 (29.7)	0.95
**Smokers [n (%)]**	25 (41)	26 (39.4)	36 (54.5)	33 (53.2)	33 (44.6)	0.32
**Hyperlipidemia [n (%)]**	42 (68.9)	41 (62.1)	46 (69.7)	39 (62.9)	46 (62.2)	0.5
**Hypertension [n (%)]**	28 (45.9)	33 (50)	25 (37.9)	17 (27.4)	26 (35.1)	**0.023**
**MI [n (%)]**	31 (51.7)	31 (47)	35 (53.8)	38 (61.3)	45 (60.8)	0.09

BMI, body mass index; MI, myocardial Infarction; P value for discrete variables based on Chi-Square Test and for continuous variables based on ANOVA. Discrete and continuous variables data are presented as number (percent) and mean (SD). High educational attainment was defined as educational level more than 12 years.

**Table 2:** Baseline characteristics of female patients candidate for CABG according to TAC quintiles.

Quintiles of TAC score						
Variable	1^st^ quintiles	2^nd^ quintiles	3^rd^ quintiles	4^th^ quintiles	5^th^ quintiles	P value
	N = 28	N = 24	N = 24	N = 28	N = 15	
**Age (y)**	61.79± 8.35	57.33 ±6.68	59.21± 6.11	58.57 ± 6.81	60.60± 9.55	0.25
**BMI (kg/m**^**2**^**)**	28.89± 5.38	31.30± 4.33	28.77 ±4.39	29.62± 4.79	28.57±3.78	0.27
**Diabetic [n (%)]**	19 (67.9)	18 (75)	16 (66.7)	19 (67.9)	8 (53.3)	0.37
**Smokers [n (%)]**	1 (3.6)	0	0	2 (7.1)	0	0.81
**Hyperlipidemia [n (%)]**	25 (89.3)	23 (95.8)	21 (87.5)	23 (82.1)	14 (93.3)	0.61
**Hypertension [n (%)]**	18 (64.3)	16 (66.7)	20 (83.3)	21 (75)	10 (66.7)	0.48
**MI [n (%)]**	8 (28.6)	11 (45.8)	13 (54.2)	10 (35.7)	3 (20)	0.71

BMI, body mass index; MI, myocardial Infarction; P value for discrete variables based on Chi-Square Test and for continuous variables based on ANOVA. Discrete and continuous variables data are presented as number (percent) and mean (SD). High educational attainment was defined as educational level more than 12 years.

**Table 3:** Odd’s ratio (OR) and confidence interval (CI) for the association between TAC and biochemical variables in male patients candidate for CABG.

Quintile of TAC score					
Variable	1^st^ quintile	2^nd^ quintile	3^rd^ quintile	4^th^ quintile	5^th^ quintile
	N = 61	N = 66	N = 66	N = 62	N = 74
**Serum vitamin D (ng/ml)**	1 (Ref.)	**1.03 (1.02–1.07)**	1.02 (0.99–1.05)	0.99 (0.96–1.03)	**1.02 (0.98–1.05)**
**HbA**_**1**_**C (%)**	1 (Ref.)	1.08 (0.87–1.35)	1.01 (0.81–1.27)	1.12 (0.90–1.39)	1.06 (0.86–1.32)
**TC (mg/dl)**	1 (Ref.)	0.98 (0.95–1.02)	1.02 (0.98–1.06)	0.98 (0.95–1.09)	**0.97 (0.94–1.003)**
**TG (mg/dl)**	1 (Ref.)	1.00 (0.99–1.002)	0.99 (0.98–1.002)	1.01 (0.99–1.01)	1.01 0.99–1.009)
**LDL (mg/dl)**	1 (Ref.)	1.02 (0.98–1.05)	0.98 (0.94–1.02)	1.01 (0.97–1.04)	1.02 (0.99–1.06)
**HDL (mg/dl)**	1 (Ref.)	1.01 (0.95–1.07)	0.97 (0.92–1.044)	1.02 (0.96–1.08)	1.04 (0.98–1.10)
**HCT (%)**	1 (Ref.)	**0.90 (0.82–0.99)**	**0.86 (0.78–0.95)**	**0.83 (0.75–0.93)**	**0.89 (0.81–0.99)**
**Albumin (g/dL)**	1 (Ref.)	**2.94 (0.87–9.92)**	2.55 (0.75–8.68)	**3.43 (0.95–12.32)**	**4.18 (1.27–13.77)**
**Creatinine (mg/dL)**	1 (Ref.)	0.84 (0.15–4.69)	0.86 (0.15–4.96)	0.27 (0.04–1.9)	1.03 (0.19–5.52)
**BUN (mg/dL)**	1 (Ref.)	0.98 (0.95–1.02)	0.97 (0.93–1.01)	0.99 (0.96–1.03)	0.98 (0.95–1.02)
**Lipoprotein (a) (mg/dL)**	1 (Ref.)	0.99 (0.98–1.02)	1.001 (0.98–1.02)	1.01 (0.98–1.02)	0.99 (0.98–1.01)
**CRP (mg/dL)**	1 (Ref.)	1.02 (0.99–1.13)	0.92 (0.80–1.06)	1.01 (0.89–1.12)	1.02 (0.92–1.12)

CI, confidence interval; TAC, total dietary antioxidant capacity; Hb, hemoglobin; TC, total cholesterol; TG, triglyceride; LDL, low density lipoprotein cholesterol; HDL, high density lipoprotein cholesterol; HCT, hematocrit; BUN, blood urea nitrogen; Lp, lipoprotein; CRP, C-reactive protein. The multivariate multinomial logistic regression was used for estimation of ORs and confidence interval (CI) with adjustment for the confounding effects of age, gender, BMI and presence of diabetes and myocardial infarction. Bold digits indicate statistically significant values as P < 0.05.

**Table 4:** Odd’s ratio (OR) and confidence interval (CI) for the association between TAC and biochemical variables in female patients candidate for CABG.

Quartiles of TAC score					
Variable	1^st^ quartile	2^nd^ quartile	3^rd^ quartile	4^th^ quartile	5^th^ quintile
	N = 24	N = 24	N = 28	N = 15	N = 74
**Vitamin D (ng/ml)**	1 (Ref.)	0.94 (0.90–0.99)	0.99 (0.95–1.04)	0.99 (0.95–1.04)	0.94 (0.89–0.99)
**HbA**_**1**_**C (%)**	1 (Ref.)	1.17 (0.76–1.81)	0.93 (0.59–1.43)	0.97(0.64–1.47)	0.80 (0.48–1.34)
**TC (mg/dl)**	1 (Ref.)	1.37 (0.84–2.24)	1.31 (0.79–2.15)	1.35 (0.83–2.21)	1.31 (0.78–2.21)
**TG (mg/dl)**	1 (Ref.)	0.93 (0.85–1.03)	0.95 (0.85–1.05)	0.94 (0.85–1.03)	0.94 (0.85–1.05)
**LDL (mg/dl)**	1 (Ref.)	0.71 (0.45–1.68)	0.75 (0.45–1.24)	0.73 (0.45–1.20)	0.75 (0.44–1.26)
**HDL (mg/dl)**	1 (Ref.)	0.71 (0.44–1.61)	0.80 (0.48–1.31)	0.75 (0.46–1.23)	0.73 (0.44–1.26)
**HCT (%)**	1 (Ref.)	1.11 (0.89–1.40)	1.01 (0.81–1.24)	1 (0.80–1.25)	1.05 (0.82–1.35)
**Albumin (g/dL)**	1 (Ref.)	3.85 (0.45–33.20)	0.2 (0.02–1.55)	0.83 (0.13–5.53)	1.27 (0.12–12.16)
**Creatinine (mg/dL)**	1 (Ref.)	0.22(0.002–20.82)	**0.01 (0–1.99)**	0.58 (0.01–32.15)	0.02 (0–3.58)
**BUN (mg/dL)**	1 (Ref.)	1.07 (0.99–1.15)	1.07 (0.98–1.15)	1.03 (0.95–1.03)	1.01 (0.92–1.10)
**Lp (a) (mg/dL)**	1 (Ref.)	1.01 (0.98–1.03)	0.99 (0.96–1.07)	1.01(0.99–1.03)	1.02 (0.99–1.04)
**CRP (mg/dL)**	1 (Ref.)	1.02 (0.86–1.2)	1.11 (0.94–1.31)	0.84 (0.63–1.12)	0.84 (0.55–1.28)

CI, confidence interval; TAC, total dietary antioxidant capacity; Hb, hemoglobin; TC, total cholesterol; TG, triglyceride; LDL, low density lipoprotein cholesterol; HDL, high density lipoprotein cholesterol; HCT, hematocrit; BUN, blood urea nitrogen; Lp, lipoprotein; CRP, C-reactive protein. The multivariate multinomial logistic regression was used for estimation of ORs and confidence interval (CI) with adjustment for the confounding effects of age, gender, BMI and presence of diabetes and myocardial infarction. Bold digits indicate statistically significant values as P < 0.05.

## Discussion

According to the results of the current work, high total antioxidant capacity of the diet was associated with the lower prevalence of hypertension, lower hematocrit and higher serum albumin concentrations in male candidate for CABG. Moreover, higher serum vitamin D was also accompanied with higher TAC in males which was a novel finding by itself. In females, however, only lower serum creatinine was associated with high TAC values.

In recent years, there are emerging evidences supporting the role of oxidative stress in the pathogenesis of hypertension [39–41]. Numerous studies even demonstrated that hypertension is the result of increase reactive oxygen species (ROS) [42–44] and antioxidant therapy relieves hypertension in animal or human models. It has been proposed that several possible underlying mechanisms of the role of oxidative stress in the pathophysiology of hypertension are increased reactive aldehyde, methylglyoxal, disrupted vascular calcium channels, enzymes, [45] oxidation of low-density lipoproteins, primarily by oxygen-free radicals [46], reduced bioactivity of NO and the activation of NAD(P)H oxidase in long term development of hypertension [47]. Several studies also have reported the negative association between dietary antioxidants intakes and development of hypertension; in the study by Kumar et al dietary antioxidant vitamin intake in hypertensive individuals were lower than normotensive individuals [48]. In another study by Rodriguez-Iturbe et al [49] antioxidant-enriched diet reduced the renal interstitial inflammation and improved hypertension in spontaneously hypertensive rats. In the current study, for the first time, we revealed the possible protective role of total dietary antioxidant capacity as a global measure of antioxidant potential of diet against hypertension. Numerous previous studies have revealed that elevated hematocrit concentration is a well-known risk factor of cardiovascular disease [50, 51]; these associations were gender-specific with more pronunciation in men possibly because of higher blood hematocrit concentrations in men; however several other studies also revealed the higher risk of CVD in women by elevated hematocrit concentrations [52]. Increased hematocrit concentrations is involved in increased CVD risk via: increased blood viscosity and raising peripheral resistance, reducing blood flow and perfusion [53], activation of platelets by releasing adenosine diphosphate (ADP) [54] and increased oxidative stress and lipid peroxidation by accumulated iron [55]. In our study, higher TAC of diet was associated with lower hematocrit concentrations; a valuable finding confirming the possible protective role of dietary antioxidants in reducing HCT and reducing the risk of CVD.

The direct association between higher serum albumin concentrations and high total dietary antioxidant capacity is also an evidence of the protective role of dietary antioxidants against CVD risk factors; in has been clarified that reduced serum albumin concentration is a known risk factor of cardiovascular events [56] and measuring its concentration could be a valuable diagnostic marker in the risk prediction of CVD [57]. Reduced serum albumin concentrations is associated with increased mortality from CVD, stroke and chronic heart disease [58]. Albumin has major antioxidant capabilities; it is a major antioxidant in plasma, accounting for 70% of free radical-trapping activity of serum due to its thiol group and high plasma concentration [59]. In addition, the ligand-binding ability of albumin contributes to its antioxidant ability because it limits the oxidation of LDL from copper or hydroxyl radical production from iron reaction with hydroperoxide [60]. It has been reported that increased serum albumin per unit is associated with a 12% reduced risk of CVD incidence over a three-year period [61]. Our study demonstrated that increased dietary antioxidant capacity increases serum albumin concentrations in men confirming its antioxidant capabilities. Another finding of the current study was increased serum vitamin D concentration in parallel of increased TAC. Vitamin D exerts its antioxidant actions by reduced peroxidation of lipids, suppression of nicotine amide adenine dinucleotide phosphate enzyme expression and inhibition of the AGEs accumulation in the cardiac vessels [62, 63]. Moreover, vitamin D reduces impairment in endothelial tissue after hydrogen superoxide induced stress and prevents ROS production by inhibiting MEKs/ERKs/SIRT-1 axis switching [64, 65]. Vitamin D exerts its cardio-protective effects via anti-oxidant and anti-inflammatory roles which have been clarified previously [66].

In addition, in men also high TAC scores were associated with lower TC concentrations. Dietary and supplementary antioxidant therapy is associated with lipid and cholesterol lowering effects as indicated in numerous previous studies [67, 68]. However, our study revealed an inverse association between dietary antioxidant capacity and TC as a major CVD risk factor.

Several limitations of the current study should also be addressed; the self-reported dietary information, not measuring indices of central adiposity were potential limitations of the current study. The study’s relatively large sample size and inclusion of multiple confounders in the statistical model are potent strengths of the current study. Moreover, this is the first study evaluated the association between TAC and CVD risk factors in patients candidate for CABG.

## Conclusion

In conclusion, our study, for the first time revealed that total dietary antioxidant capacity as a global measure of dietary antioxidant has a potential association with the major cardiovascular risk factors including HCT, TC, albumin and serum vitamin D in patients’ candidate for CABG. These associations were gender-specific with more pronunciation in men. CABG role as a trigger of oxidative stress and increased free radicals has been reported before, so, the findings of the current study has a major beneficial applications in advising these patients to increase their dietary antioxidant capability to reduce the CVD risk factors.

## Supporting information

S1 Dataset (SAV)

## Abbreviations

TAC: total dietary antioxidant capacity
ANOVA: analysis of variance
CAD: coronary artery disease
CVD: cardiovascular disease
HDL: high density lipoprotein cholesterol
LDL: low density lipoprotein cholesterol
TC: total cholesterol
LP: lipoprotein
CABG: coronary artery bypass grafting surgery
CRP: C-reactive protein
